# The effects of exercise on dynamic sleep morphology in healthy controls and patients with chronic fatigue syndrome

**DOI:** 10.1002/phy2.152

**Published:** 2013-11-13

**Authors:** Akifumi Kishi, Fumiharu Togo, Dane B Cook, Marc Klapholz, Yoshiharu Yamamoto, David M Rapoport, Benjamin H Natelson

**Affiliations:** 1 Division of Pulmonary, Critical Care and Sleep Medicine, Department of Medicine, New York University School of MedicineNew York City, New York; 2 Japan Society for the Promotion of ScienceTokyo, Japan; 3 Educational Physiology Laboratory, Graduate School of Education, The University of TokyoTokyo, Japan; 4 William S. Middleton Memorial Veterans Hospital and Department of Kinesiology, University of Wisconsin School of EducationMadison, Wisconsin; 5 Department of Medicine, Rutgers-New Jersey Medical SchoolNewark, New Jersey; 6 Pain & Fatigue Study Center, Department of Neurology, Rutgers-New Jersey Medical SchoolNewark, New Jersey

**Keywords:** Chronic fatigue syndrome, cumulative duration distributions, exercise, sleep stage dynamics, transition probability

## Abstract

Effects of exercise on dynamic aspects of sleep have not been studied. We hypothesized exercise altered dynamic sleep morphology differently for healthy controls relative to chronic fatigue syndrome (CFS) patients. Sixteen controls (38 ± 9 years) and 17 CFS patients (41 ± 8 years) underwent polysomnography on baseline nights and nights after maximal exercise testing. We calculated transition probabilities and rates (as a measure of relative and temporal transition frequency, respectively) between sleep stages and cumulative duration distributions (as a measure of continuity) of each sleep stage and sleep as a whole. After exercise, controls showed a significantly greater probability of transition from N1 to N2 and a lower rate of transition from N1 to wake than at baseline; CFS showed a significantly greater probability of transition from N2 to N3 and a lower rate of transition from N2 to N1. These findings suggest improved quality of sleep after exercise. After exercise, controls had improved sleep continuity, whereas CFS had less continuous N1 and more continuous rapid eye movement (REM) sleep. However, CFS had a significantly greater probability and rate of transition from REM to wake than controls. Probability of transition from REM to wake correlated significantly with increases in subjective fatigue, pain, and sleepiness overnight in CFS – suggesting these transitions may relate to patient complaints of unrefreshing sleep. Thus, exercise promoted transitions to deeper sleep stages and inhibited transitions to lighter sleep stages for controls and CFS, but CFS also reported increased fatigue and continued to have REM sleep disruption. This dissociation suggests possible mechanistic pathways for the underlying pathology of CFS.

## Introduction

Exercise is assumed to affect the subsequent night of sleep through alternations of physiologic systems (Uchida et al. 2012). Although it is often believed that exercise improves sleep, experimental sleep studies based upon polysomnography (PSG) have not shown substantial sleep-promoting effects of exercise (Youngstedt 2005). However, modest effects of acute exercise on sleep have been reported. According to a recent review paper on exercise and sleep (Uchida et al. 2012), which includes two meta-analytic studies (Kubitz et al. 1996; Youngstedt et al. 1997), consistent findings of the effects of acute exercise on sleep were increased total sleep time (TST), increased slow-wave sleep (SWS) duration, and decreased rapid eye movement (REM) sleep duration. In addition, acute exercise is also reported to shorten sleep latency, increase REM latency, and increase stage 2 sleep (Kubitz et al. 1996; Youngstedt et al. 1997). These findings are consistent with an increase in sleep pressure after exercise. Since many experimental studies have been limited to healthy sleepers whose sleep is already very good, ceiling and/or floor effects have been suggested as a reason that only modest effects of exercise on sleep have been reported (Youngstedt 2003).

Chronic fatigue syndrome (CFS) is a medically unexplained condition characterized by persistent or relapsing fatigue lasting at least 6 months, which substantially reduces normal activity (Fukuda et al. 1994). In addition to severe fatigue, one of the most common ancillary symptoms of CFS patients is “unrefreshing sleep” (Unger et al. 2004; Reeves et al. 2006). A disabling and characteristic feature of CFS is that even minimal exertion produces a worsening of symptoms, that is, subjective complaints relating to fatigue/sleep/cognitive function (King and Jason 2005). We have recently performed a study investigating the influence of acute exercise on sleep in CFS and reported that while exercise did not worsen sleep in patients with CFS, exercise actually improved it in healthy controls (Togo et al. 2010). These data do not answer the question as to why patients with CFS report worsening of their symptoms after exercise. Newer approaches at assessing sleep do exist which may be useful in determining why CFS patients do have a sleep problem after exercise. Understanding the reason for this problem will be important in understanding the pathophysiology of CFS as well as in getting insights into better treatment of sleep problems in CFS.

One such new approach evaluates dynamic aspects of sleep, which are composed of sequential transitions between sleep stages and continuous runs of each sleep stage (defined as periods of continuous epochs of each sleep stage separated by one of the other stages). These can be quantified by transition probabilities and rates between sleep stages and continuity analysis of each sleep stage (Kishi et al. 2008, 2011a,b), and data exist that this method can provide useful information beyond traditional sleep variables (Rechtschaffen and Kales 1968; Iber et al. 2007) as well as help better understand the effects of illness on sleep. For instance, sleep continuity analysis has revealed a difference between patients with mild sleep-disordered breathing (SDB) and controls where traditional sleep stage variables, such as TST, sleep efficiency, and the number of sleep stage shifts, did not detect any difference (Norman et al. 2006). Also, transition analysis has revealed a difference between patients with CFS with or without fibromyalgia where traditional sleep variables did not detect any difference (Kishi et al. 2011b).

While there is growing interest in understanding dynamic aspects of sleep (Lo et al. 2002, 2004; Comte et al. 2006; Kishi et al. 2008), that interest has not yet extended to assessing the effects of exercise. Given the utility of dynamic transition analysis of sleep stages, we hypothesized that there might be effects of exercise on dynamic aspects of sleep not captured by usual static sleep evaluations. In this study, we investigate the effects of exercise on dynamic aspects of sleep, that is, transition probabilities and rates between sleep stages and cumulative duration distributions (as a measure of continuity) of each sleep stage and sleep as a whole, in our previously reported study population of healthy controls and patients with CFS (Togo et al. 2010). We hypothesized that exercise would affect dynamic sleep morphology differently in healthy controls and patients with CFS. We expected healthy controls to report reduced fatigue and sleepiness with improvements of dynamic sleep morphology after exercise in contrast to the patient group for whom we postulated more symptoms to explain their common complaints of “unrefreshing sleep” and symptom worsening after effort.

## Methods

### Subjects

The subjects were 33 women, 17 with CFS (age: 41 ± 8 years; mean ± SD) and 16 healthy controls (38 ± 9 years), whose traditional sleep variables have previously been reported (Togo et al. 2010). Subjects with CFS were either physician-referred or self-referred in response to media reports about our research. Healthy controls were acquaintances of patients or responded to recruitment flyers. All of these subjects were habituated to the sleep laboratory and had been screened for sleep disorders by a previous night of diagnostic PSG (Togo et al. 2008), and all were negative for sleep disorders in the form of restless leg syndrome or obstructive sleep apnea. Patients taking antidepressants, opiates, steroids, hypnotics, and other sedatives, including benzodiazepines were excluded from further study. The patients all fulfilled the 1994 case definition for CFS (Fukuda et al. 1994) and thus had neither any medical explanation for their symptoms based on history, physical examination, and blood tests to rule out other disorders nor did they have any serious psychiatric diagnoses, including schizophrenia, eating disorders, substance abuse, or bipolar disorder, as determined by psychiatric diagnostic interview (the computerized version of the Diagnostic Interview Schedule [DIS-IV]) (Robbins et al. 2000). Controls reported not exercising more than once a week, having either excellent or good health, and had normal findings upon examination with normal results on blood tests. Because major depressive disorder is known to be associated with frequent sleep-electroencephalographic changes (Ford and Cooper-Patrick 2001), we also used the DIS to confirm that no subject with major depressive disorder was included. To further reduce variability, menstrual subjects were all studied in the follicular phase of their menstruating cycles. All the subjects provided their informed consent, approved by the New Jersey Medical School's Institutional Review Board, to participate in this research.

### Experimental Procedures

Following instructions to refrain from alcohol and caffeine ingestion and to avoid engaging in prolonged and/or strenuous exercise in the daytime before study nights, the subjects underwent two nocturnal PSG studies to provide the data reported here. The subjects went to bed at their usual bedtime and awoke the next morning between 07:15 and 08:00.

### Polysomnography

Within 6 months of their habituation session in the sleep laboratory, subjects returned to the sleep laboratory for two more nights of study during which they were instrumented to allow recording of electroencephalogram (C3/A2, O1/A2, and FZ/A2), electrooculogram, submental electromyogram, and a lead II electrocardiogram (ECG). The first of these nights was used as a control for the final night before which subjects performed the maximal exercise test detailed below. Exercise was always performed before the final night to avoid a carryover effect to the subsequent night. On these two nights, subjects had indwelling venous catheters from which blood was sampled remotely without disturbing the subject three times during the course of the night. Blood was collected to test the hypothesis that cytokine dysregulation is important in the genesis of the disorder and we used exercise and sleep deprivation to test this hypothesis comparing cytokines in these conditions to those found during a baseline sleep night (these data have been reported elsewhere [Nakamura et al. 2010, 2013]). Sleep was scored every 30 sec by a single scorer according to the standard criteria of Rechtschaffen and Kales (R&K) (Rechtschaffen and Kales 1968).

### Exercise

A maximal exercise test was performed on an electronically braked cycle ergometer (Lode Corival, Groningen, the Netherlands) during the late afternoon. The seat height and toe clips were adjusted to the desired fit of the subject. The exercise test began with a 3-min unloaded warm-up. After warm-up, exercise began at 20 W. Exercise intensity was then increased by 5 W every 20 sec until volitional exhaustion or the point where the subject could no longer maintain the prescribed minimum pedal rate (60 rpm). During the exercise test, measurements of oxygen consumption 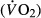, carbon dioxide production 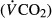, and expired ventilatory volume 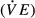 were obtained breath-by-breath using a MedGraphics Gas Exchange and Pulmonary Function System (Medgraphics Ultima CPX System with Breeze Suite Software; Medical Graphics Corporation, St. Paul, MN). Heart rate (HR) was monitored during exercise by ECG using a Quinton Q4000 (Quinton Instruments, Seattle, WA). Because of technical difficulties, HR data were not acquired on three CFS participants and two control participants. Participants were encouraged during the test to continue as long as possible and to give their best possible effort. Acceptable effort was determined as achieving 80% of age-predicted maximum HR and/or a respiratory exchange ratio (RER) ≥1.1.

### Subjective test

A visual analog scale (0–15.5 cm) was used to estimate perceived fatigue, pain, sleepiness, feeling blue, and anxiety before and after each PSG recording. Visual analog scales have consistently been shown to provide valid measures of subjective feelings (McCormack et al. 1988; Togo et al. 2008).

### Data analysis

In this study, sleep stages 3 and 4, scored using R&K criteria (Rechtschaffen and Kales 1968), were combined into a single-stage N3 to approximate the new scoring guidelines developed by the American Academy of Sleep Medicine (Iber et al. 2007).

We used normalized transition probabilities to characterize sleep stage transitions (Kishi et al. 2008, 2011a,b). Each normalized transition probability between wake, REM, N1, N2, and N3 was calculated by dividing the number of transitions from a specific stage to one of the other stages by the total number of the transitions from that specific stage to another stage, and multiplying it by 100. We also used the transition rate per hour, which was calculated by dividing the number of transitions between sleep stages by the total duration (h) of the origin stage of that transition, to complement the interpretation of the result. These transition statistics were first calculated (normalized) within the individuals and then averaged across subjects in each group.

The probability densities for durations for each sleep stage and sleep as a whole (i.e., continuous runs of each sleep stage and sleep as a whole) were analyzed by pooling those of all the individuals in each group: we considered pooling the data since the number of durations of each of these classes of transition was small due to the brevity of one night of sleep. Cumulative duration distributions were calculated as 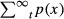, where *p*(*t*) is the probability density function of duration *t*. In this study, cumulative duration distributions were presented in linear plot for each sleep stage and sleep as a whole (Fig. 2 for healthy controls and Fig. 3 for CFS patients), as well as in double logarithmic plot for wake and N3 and in semilogarithmic plot for sleep as a whole, N1, N2, and REM (Fig. 2, insets, for healthy controls and Fig. 3, insets, for CFS patients), for the purpose of making the differences of distributions more visible (Kishi et al. 2011b).

### Statistical analysis

Differences in the number of continuous runs of each sleep stage, transition probabilities for subjects who had nonzero transition probabilities and transition rates per hour were assessed using a two-way mixed factorial analysis of variance (ANOVA). Post hoc analyses used paired *t*-tests for within-subjects comparisons (i.e., between conditions; baseline vs. postexercise) and nonpaired *t*-tests for between-subjects comparisons (i.e., between groups; healthy controls vs. CFS patients) with Bonferroni corrections. In addition, differences in transition probabilities, transition rates, and subjective feelings captured via the visual analog scale were also separately assessed using paired *t*-test for within-subject comparisons in each group since the effects of exercise on sleep in each group were of particular interest in this study.

Differences of cumulative duration distributions for each sleep stage and sleep as a whole were assessed using the log rank test and the generalized Wilcoxon test, where the former test is relatively sensitive to the later part of the difference in survival curves and the latter test is relatively sensitive to the earlier part of the difference in survival curves (Martinez and Naranjo 2010). We used both of the tests for comparisons of stage- and sleep-continuity curves since it is possible that differences appear in either of the earlier or of the later part of continuity curves. Comparisons were made between conditions (baseline vs. postexercise) in each group. To assess interindividual differences in probability distributions of durations for each sleep stage and sleep as a whole, we used the Kolmogorov–Smirnov test for all the pairs of individuals in each group.

Relationships between the specific transitions (probability and rate) and change of the subjective feelings over night (before and after sleep) were examined using partial correlation analyses controlling for conditions for each group; these were done after verifying specific patterns of transitions that might affect subjective feelings specifically in CFS. Statistical significance was accepted when *P* was less than 0.05.

## Results

### Exercise

All subjects were able to achieve at least one of the two criteria of an acceptable effort, that is, (1) achieving 80% of age-predicted maximum HR and/or (2) a RER ≥1.1, and the majority (13/17 CFS and 13/16 controls) met both criteria. CFS and control groups did not differ significantly in percent peak HR (CFS = 87 ± 6%, controls = 91 ± 7%; mean ± SD), peak RER (CFS = 1.26 ± 0.17, controls =1.30 ± 0.09; mean ± SD), or peak 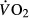 (CFS = 20.1 ±5.4 mL kg^−1^ min^−1^, controls = 24.5 ± 5.1 mL kg^−1^ min^−1^; mean ± SD) (Togo et al. 2010).

### Sleep variables

**Figure 1:**
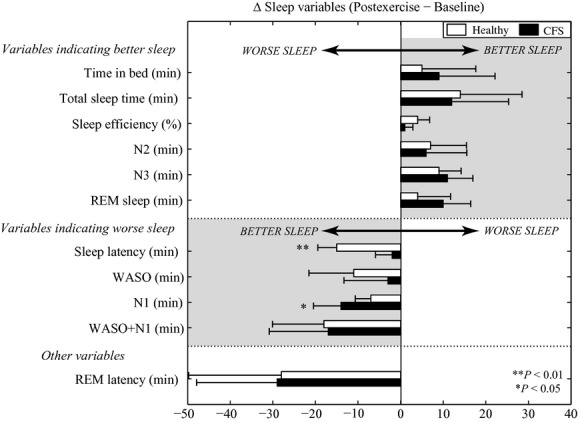
Differences of values in sleep variables between baseline night and postexercise night for healthy controls (white) and patients with CFS (black). Values at the baseline night are subtracted from those at the postexercise night. Areas colored by gray indicate improved sleep by exercise (e.g., shorter sleep latency or less wakefulness after sleep onset [WASO] would mean improved sleep). Error bars depict standard errors of means. **P < 0.01 and *P < 0.05 by paired *t*-test.

Traditional descriptive statistics of sleep parameters for healthy controls and patients with CFS on their baseline and postexercise nights have been published elsewhere (Togo et al. 2010). Differences of values in sleep variables between nights (postexercise night − baseline night) are illustrated in Figure 1. Healthy controls showed significantly decreased sleep latency on the postexercise night than on their baseline night (*P* < 0.01). Patients with CFS showed significantly decreased total duration of N1 and a significantly decreased arousal index on the postexercise night than on their baseline night (*P* < 0.05 for total N1 duration and *P* < 0.01 for arousal index). There was no other significant difference in sleep variables between baseline and postexercise nights for either group.

Two-way mixed factorial ANOVA revealed that there was a significant main effect of groups and conditions for the number of continuous runs of waking (22.8 ± 8.2 on the baseline night and 19.2 ± 7.6 on the postexercise night in healthy controls, and 29.5 ± 14.0 on the baseline night and 25.2 ± 7.3 on the postexercise night in patients with CFS; *P* < 0.05, values are mean ± SD): patients with CFS had a greater number of continuous runs of waking than controls, and exercise significantly decreased the number of continuous runs of waking. There was no other significant main effect or interaction for the number of continuous runs of each sleep stage (N1, N2, N3, and REM).

### Transition probabilities

Normalized probabilities of transitions between the five sleep stages (wake, REM, N1, N2, and N3) of healthy controls and patients with CFS on their baseline and postexercise nights are shown in Table 1.

**Table 1:** Normalized transition probabilities between sleep stages

(%)	Healthy			CFS		
	
	B	E	Δ	B	E	Δ
Transitions indicating better sleep						
N1→N2^c^	59.3	66.8*	+7.5*	66.9	64.9	−2.0
N1→N3	0.2	0.0	−0.2	0.0	0.0	0
N2→N3^b^^,^^d^	34.6	34.6	+0.1	31.1	38.0*	+6.9*
Transitions indicating worse sleep						
N1→W	20.1	13.6	−6.5‡	20.4	21.4	+1.0
N2→W	22.7	21.7	−1.0	23.6	21.5	−2.1
N2→N1	31.2	31.4	+0.2	36.9	30.8	−6.2‡
N3→W	2.8	3.1	+0.4	2.9	5.7	+2.6
N3→N1	0.7	2.2	+1.6	1.9	0.8	−1.2
R→W^a^	18.3	22.2	+3.9	33.2	28.5	−4.7
Other transitions						
W→N1	90.3	88.0	−2.3	86.8	88.3	+1.4
W→N2	7.7	9.4	+1.8	10.5	8.9	−1.7
W→N3	0.3	0.0	−0.3	0.0	0.0	0
W→R	1.7	2.5	+0.8	2.6	2.9	+0.2
N1→R^a^	20.4	19.6	−0.9	12.7	13.7	+1.0
N2→R	11.5	12.3	+0.8	8.4	9.7	+1.4
N3→N2	96.5	93.7	−3.0	95.2	93.6	−1.4*
N3→R	0.0	1.0	+1.0	0.0	0.0	0
R→N1	66.3	59.5	−6.8	46.9	55.5	+8.6
R→N2	15.5	18.1	+2.6	19.3	16.0	−3.2
R→N3	0.0	0.3	+0.2	0.7	0.0	−0.7

Normalized transition probabilities between waking (W), REM sleep (R), N1, N2, and N3 for the baseline night (B), the postexercise night (E) and the difference between conditions (Δ, postexercise − baseline) for healthy controls and patients with CFS.

a *P <* 0.05 for main effect of group.

b *P <* 0.05 for main effect of condition.

c *P <* 0.05 for interaction by ANOVA.

d *P* = 0.053 for interaction by ANOVA.

* *P* < 0.05 by paired *t*-test.

‡ *P* < 0.06 by paired *t*-test.

#### Improved patterns of transitions produced by exercise

Two-way mixed factorial ANOVA revealed that there was a significant interaction for the probability of transition from N1 to N2 (*P* < 0.05): post hoc tests revealed that exercise significantly increased transition probability from N1 to N2 in healthy controls (*P* < 0.05) but this effect was not observed in patients with CFS. There was a significant main effect of conditions for the probability of transition from N2 to N3 (*P* < 0.05): exercise significantly increased transition probability from N2 to N3. There was a trend toward an interaction for the probability of transition from N2 to N3 (*P* = 0.053): post hoc tests revealed that exercise significantly increased transition probability from N2 to N3 in patients with CFS (*P* < 0.05) but this effect was not observed in healthy controls.

When focusing on the analyses for within-subject comparisons (i.e., baseline vs. postexercise in each group) only, exercise significantly increased the probability of transition from N1 to N2 in healthy controls (*P* < 0.05) and that of transition from N2 to N3 in patients with CFS (*P* < 0.05). Also, exercise tended to decrease the probability of transition from N1 to wake in healthy controls (*P* < 0.06) and that of transition from N2 to N1 in CFS patients (*P* < 0.06).

#### Worse pattern of transitions in CFS

Two-way mixed factorial ANOVA revealed that there was a significant main effect of groups for the probability of transition from REM to wake (*P* < 0.05): patients with CFS had greater transition probability from REM to wake than healthy controls.

#### Other altered patterns of transitions in CFS

Two-way mixed factorial ANOVA revealed a significant main effect of groups for the probability of transition from N1 to REM (*P* < 0.05): patients with CFS had a smaller transition probability from N1 to REM than healthy controls.

Focusing on the analyses for within-subject comparisons only, exercise significantly decreased the probability of transition from N3 to N2 in patients with CFS (*P* < 0.05).

There was no other significant main effect or interaction, or within-subject difference in either group for transition probabilities.

### Transition rates

Rates of transitions among the five sleep stages (wake, REM, N1, N2, and N3) of healthy controls and patients with CFS on their baseline and postexercise nights are shown in Table 2.

**Table 2:** Transition rates between sleep stages per hour

(×10^−2^)	Healthy			CFS		
	
	B	E	Δ	B	E	Δ
Transitions indicating better sleep						
N1→N2	60.3	70.3	+9.9	70.6	71.9	+1.3
N1→N3	0.2	0.0	−0.2	0.0	0.0	0
N2→N3	9.0	8.6	−0.4	8.8	10.1	+1.3
Transitions indicating worse sleep						
N1→W^c^	20.0	13.6*	−6.4*	20.0	23.8	+3.7
N2→W^b^	5.1	4.7	−0.4	*6.6*	5.5	−1.1‡
N2→N1^b^	7.6	6.6	−1.0	10.6	7.8	−2.8*
N3→W	1.7	1.3	−0.4	1.4	2.5	+1.1‡
N3→N1	0.3	0.4	+0.1	0.8	0.1	−0.7‡
R→W^a^	3.6	4.0	+0.4	8.5	6.2	−2.3
Other transitions						
W→N1	60.4	59.2	−1.2	48.2	48.0	−0.3
W→N2	6.0	7.6	+1.6	4.4	6.1	+1.7
W→N3	0.2	0.0	−0.2	0.0	0.0	0
W→R	1.0	2.3	+1.4	1.8	2.0	+0.1
N1→R^a^	19.6	18.4	−1.2	12.0	13.8	+1.8
N2→R	2.5	2.7	+0.2	2.3	2.4	+0.2
N3→N2	90.8	81.8	−6.8	72.3	55.7	−14.8
N3→R	0.0	1.4	+1.5	0.0	0.0	0
R→N1	18.3	14.8	−3.5	14.1	13.9	−0.2
R→N2	2.9	2.6	−0.3	4.0	2.6	−1.4
R→N3	0.0	0.1	+0.1	0.1	0.0	−0.1

Transition rates between waking (W), REM sleep (R), N1, N2, and N3 for the baseline night (B), the postexercise night (E) and the difference between conditions (Δ, postexercise − baseline) for healthy controls and patients with CFS.

a *P <* 0.05 for main effect of group.

b *P <* 0.05 for main effect of condition.

c *P <* 0.05 for interaction by ANOVA.

* *P* < 0.05 by paired *t*-test.

‡ *P* < 0.06 by paired *t*-test.

#### Improved patterns of transitions produced by exercise

Two-way mixed factorial ANOVA revealed a significant main effect of condition for the rate of transition from N2 to wake (*P* < 0.05) and that from N2 to N1 (*P* < 0.05): exercise significantly decreased transition rates from N2 to wake and N2 to N1. There was a significant interaction for the rate of transition from N1 to wake (*P* < 0.05): post hoc tests revealed that exercise significantly decreased transition rate from N1 to wake in healthy controls (*P* < 0.05) but not in patients with CFS.

Focusing on the analyses for within-subject comparisons (i.e., baseline vs. postexercise in each group) only, exercise significantly decreased the rate of transition from N1 to wake for healthy controls (*P* < 0.05) and that of transition from N2 to N1 (*P* < 0.05) for patients with CFS. Also, exercise tended to decrease the rates of transitions from N2 to wake (*P* < 0.06) and from N3 to N1 (*P* < 0.06) for CFS patients; however, exercise tended to increase the rate of transition from N3 to wake in patients with CFS (*P* < 0.06).

#### Worse pattern of transitions in CFS

Two-way mixed factorial ANOVA revealed a significant main effect of groups for the rate of transition from REM to wake (*P* < 0.05): patients with CFS had a greater transition rate from REM to wake than healthy controls.

#### Other altered pattern of transitions in CFS

Two-way mixed factorial ANOVA revealed a significant main effect of groups for the rate of transition from N1 to REM (*P* < 0.05): patients with CFS had a smaller transition rate from N1 to REM than healthy controls.

There was no other significant main effect or interaction, or within-subject difference in either group for transition rates.

### Cumulative duration distributions

**Figure 2:**
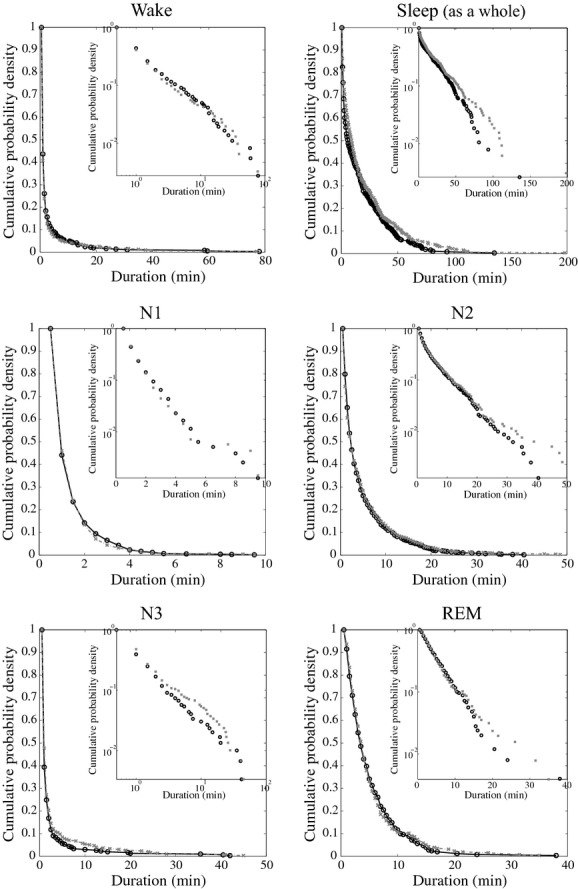
Cumulative duration distributions for wake, sleep as a whole, N1, N2, N3, and REM in healthy controls on the baseline night (black, ○) and the postexercise night (gray, ×). Insets: Cumulative duration distributions presented in double logarithmic plot for wake and N3, and in semilogarithmic plot for sleep as a whole, N1, N2, and REM, in healthy controls on the baseline night (black, ○) and the postexercise night (gray, ×).

**Figure 3:**
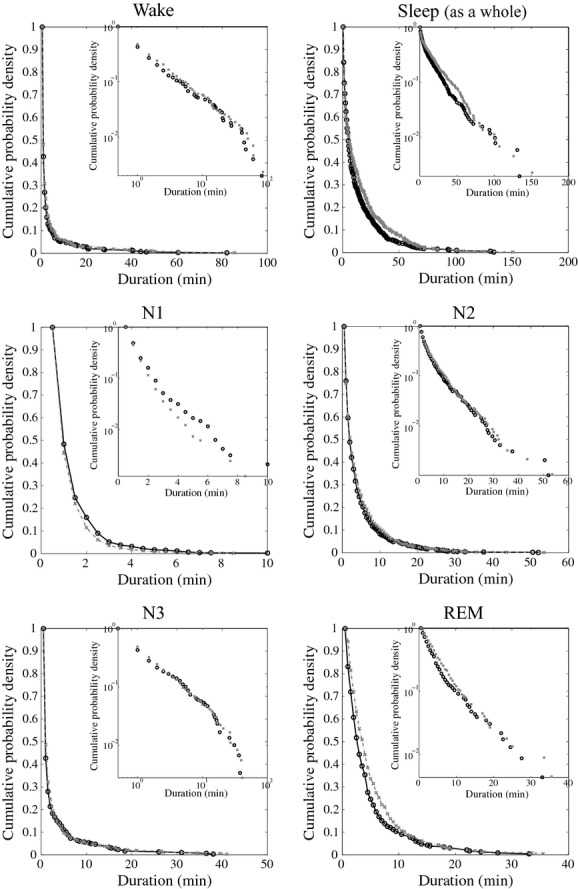
Cumulative duration distributions for wake, sleep as a whole, N1, N2, N3, and REM in patients with CFS on the baseline night (black, ○) and the postexercise night (gray, ×). Insets: Cumulative duration distributions presented in double logarithmic plot for wake and N3, and in semilogarithmic plot for sleep as a whole, N1, N2, and REM, in patients with CFS on the baseline night (black, ○) and the postexercise night (gray, ×).

Cumulative duration distributions of the five sleep stages (wake, REM, N1, N2, and N3) and sleep as a whole on baseline and postexercise nights for healthy controls and patients with CFS are shown in Figures 2, 3, respectively. To verify that data from individual recordings for wake, sleep (as a whole), REM sleep, N1, N2, and N3 periods were each drawn from the same probability distribution, we compared the probability densities pairwise among subjects for all individual sleep stages. In most cases, we were not able to show statistically significant difference in the distribution for each stage: mean ± SD of the percentages of individual pairs with positive Kolmogorov–Smirnov test for each sleep stage and condition was 14.4 ± 9.0% (range: 0.0–33.3%) for healthy controls and 16.7 ± 8.5% (range: 3.7–31.6%) for patients with CFS. This analysis supported our pooling of the data from all subjects in each group in the analysis of duration distributions for each sleep stage.

#### Improved continuity of sleep stages and/or sleep by exercise

For healthy controls, the cumulative duration distributions for sleep showed a significant shift toward longer runs of durations on the postexercise night than on the baseline night (*P* < 0.05 by both of the log rank test and the generalized Wilcoxon test). For patients with CFS, the cumulative duration distributions for REM sleep showed a significant shift toward longer runs of duration on the postexercise night than on the baseline night (*P* < 0.05 by the log rank test and *P* < 0.01 by the generalized Wilcoxon test), and the cumulative duration distributions for N1 showed a significant shift toward shorter runs of durations (*P* < 0.01 by the log rank test and *P* < 0.05 by the generalized Wilcoxon test). There was no other significant difference in continuity of sleep stages or sleep as a whole for both groups.

### Subjective feelings

**Figure 4:**
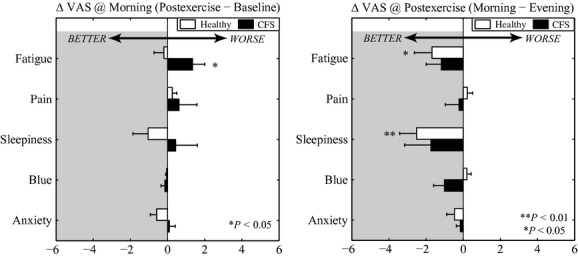
Differences of values in subjective feelings measured by visual analog scale (VAS) in the morning between baseline night and postexercise night (left panel; values at the baseline night are subtracted from those at the postexercise night) and differences of values in subjective feelings measured by VAS between before and after sleep (i.e., between evening and morning) on the postexercise night (right panel; values in the evening are subtracted from those in the morning on the postexercise night) for healthy controls (white) and patients with CFS (black). Areas colored by gray indicate improved subjective feeling by exercise. Error bars depict standard errors of means. **P < 0.01 and *P < 0.05 by paired *t*-test.

Subjective fatigue, pain, sleepiness, and feeling blue of healthy controls and patients with CFS before and after sleep (i.e., evening and morning) on the baseline and postexercise nights have been published elsewhere (Togo et al. 2010). Patients with CFS reported more anxiety in the morning after their postexercise night than healthy controls (*P* < 0.05) but not after the normal sleep night. Differences of subjective feelings (fatigue, pain, sleepiness, feeling blue, and anxiety) between the evening and the morning on the postexercise night (morning − evening) and between the morning on the baseline night and the morning on the postexercise night (postexercise night − baseline night) are illustrated in Figure 4. Healthy controls reported significantly decreased sleepiness and fatigue in the morning than in the evening on their postexercise night (*P* < 0.01 for sleepiness and *P* < 0.05 for fatigue). Patients with CFS reported significantly increased fatigue in the morning on the postexercise night than in the morning on the baseline night (*P* < 0.05). There was no other significant difference in the subjective feelings related with exercise for both group.

#### Relationship between specific transitions and subjective feelings

For those transitions showing a main effect of groups, that is, transitions from REM to wake and those from N1 to REM, we looked for relationships between those transitions and the change of the subjective feelings over night for each group. Specifically, we examined the relationships between transition variables, that is, transition probabilities and rates from REM to wake and from N1 to REM, and change of subjective feelings (fatigue, pain, sleepiness, feeling blue, and anxiety) over night for each group.

**Figure 5:**
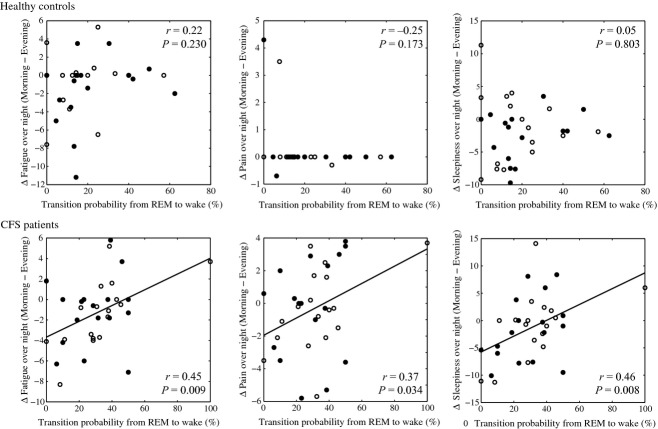
Relationships between transition probability from REM to wake and change of the subjective fatigue, pain, and sleepiness over night in healthy controls and patients with CFS. Open circles (○) represent data for the baseline night and closed circles (•) represent data for the postexercise night.

The relationship between the probability of transition from REM to wake and change of fatigue, pain, or sleepiness over night for each group is shown in Figure 5. There was a significant correlation between probability of transition from REM to wake and change of subjective fatigue, pain, or sleepiness over night in patients with CFS controlling for condition (*r* = 0.45, *P* = 0.009 for fatigue, *r* = 0.37, *P* = 0.034 for pain, and *r* = 0.46, *P* = 0.008 for sleepiness); in contrast, this correlation was not seen in healthy controls (*r* = 0.22, *P* = 0.230 for fatigue, *r* = −0.25, *P* = 0.173 for pain, and *r* = 0.05, *P* = 0.803 for sleepiness). The significant correlation between the probability of transition from REM to wake and the change of subjective fatigue or sleepiness over night in patients with CFS held true when one extreme data point, showing 100% of transition probability from REM to wake, was excluded from the analysis (*r* = 0.37, *P* = 0.039 for fatigue and *r* = 0.43, *P* = 0.014 for sleepiness); the probability of transition from REM to wake did not correlate significantly with change of subjective pain over night when the extreme data point was excluded from the analysis (*r* = 0.27, *P* = 0.140). There was no significant correlation between probability of transition from REM to wake and change of subjective feeling blue or anxiety for either healthy controls or patients with CFS.

In addition, there was no significant correlation between rate of transition from REM to wake and change of subjective fatigue, pain, or sleepiness over night in healthy controls (*r* = 0.07, *P* = 0.684 for fatigue, *r* = −0.20, *P* = 0.277 for pain, and *r* = −0.01, *P* = 0.965 for sleepiness) and patients with CFS (*r* = 0.23, *P* = 0.195 for fatigue, *r* = −0.06, *P* = 0.759 for pain, and *r* = 0.20, *P* = 0.262 for sleepiness) controlling for condition. There was no significant correlation between rate of transition from REM to wake and change of subjective feeling blue or anxiety for either healthy controls or patients with CFS.

There was no significant correlation between transition variables (probability and rate) from N1 to REM and change of any of the subjective feelings for both groups.

## Discussion

In this study, exercise promoted transitions to deeper sleep stages as well as inhibited transitions to lighter sleep stages, suggesting increased sleep pressure and/or improved quality of sleep after exercise. Specifically, healthy controls showed a significantly greater probability of transition from N1 to N2 and a lower rate of transition from N1 to wake after exercise than on their baseline, and patients with CFS showed a significantly greater probability of transition from N2 to N3 and a lower rate of transition from N2 to N1 after exercise than on their baseline. Continuity of sleep in the controls improved after exercise, while in patients with CFS continuity of N1 worsened and continuity of REM sleep improved after exercise. Despite their improvement in overall quality of sleep after exercise, CFS patients had a significantly greater probability and rate of transition from REM to wake at both baseline and postexercise night compared to healthy controls, and they reported more fatigue in the morning after exercise than on their baseline morning. Correlation analysis found that the higher the probability of transition from REM to wake, the greater the increase in fatigue overnight in patients with CFS, but not in healthy controls. These results suggest that, while exercise has positive effects on dynamic aspects of sleep for both groups, the sustained feeling of increased fatigue in CFS could be due to disruption of REM sleep, which we have previously shown to occur in CFS (Kishi et al. 2008). Our additional analyses showing positive and significant relations between transition probability from REM to wake and an increase in fatigue as well as pain and sleepiness over night suggest that the abnormality in REM in CFS may reflect an underlying pathophysiologic mechanism.

This study was the first to evaluate the effects of exercise on dynamic aspects of sleep, and this approach appears to be more sensitive in finding differences than standard R&K analyses. According to the assessment based on traditional sleep variables, the only significant changes found after exercise were a decrease of sleep latency for healthy controls and a decrease of N1 sleep for patients with CFS – that is, modest effects. Our analyses focusing on the dynamic aspects of sleep yielded more informative results for the effect of exercise on sleep – showing the capability of dynamic transition analysis to better detect alternations of sleep. Our results indicate exercise affects dynamic sleep morphology similarly for healthy controls and patients with CFS but with some evidence for even more improvement in the patient group. We interpret the increased probability of transitions from N2 to N3 in patients as better sleep than the increased probability of transitions from N1 to N2 for healthy controls because deeper sleep is known to be associated with improved daytime functioning. This difference might have emerged due to ceiling/floor effects of sleep (Youngstedt 2003) in that there could be more room for improvement for patients with CFS than for healthy controls. However, finding that exercise promotes transitions to deeper sleep stages in CFS than in controls points to the need to move studies designed to understand the effects of exercise to patients with sleep complaints rather than continuing to study healthy people who are good sleepers (Youngstedt et al. 1997; Youngstedt 2005).

Patients with CFS often report that exertion produces dramatic symptom worsening (King and Jason 2005). We have recently reported that exercise does not exacerbate sleep disturbance in CFS, based upon the evaluation using traditional sleep variables (Togo et al. 2010). Our reanalysis done here, focusing on the dynamic aspects of sleep, found a similar result. The patients, however, reported more fatigue in the morning after exercise than in the morning following the baseline night, while healthy controls showed significant improvement in sleepiness and fatigue following a night of sleep after exercise compared to the evening after exercise. This is consistent with a previous finding that CFS symptoms worsen several days after maximal exercise (Yoshiuchi et al. 2007); on the other hand, there is evidence that a course of chronic mild to moderate exercise – that is “graded exercise therapy” – improves subjective symptoms in CFS (Edmonds et al. 2004; Chambers et al. 2006). Patients get better on a chronic exercise regimen but report syndromic worsening with decreased activity following a burst of acute exertion (Sisto et al. 1998). In this study, because dynamic sleep morphology after acute exercise was improved in CFS as much as in healthy controls, one can conclude that disrupted sleep does not explain symptom worsening after exertion in CFS.

We found positive and significant correlations among the transition probability from REM to wake and an increase in subjective fatigue, pain, and sleepiness overnight for patients with CFS. The change of fatigue and sleepiness in CFS, however, did not correlate significantly with the transition rate from REM to wake. A fundamental difference between transition probability and transition rate lies in whether the metric takes account of other patterns of transition (from the same stage). Transition probability represents relative frequency of transitions with focus on the *direction* of the transition, while transition rate simply quantifies the degree of the fragmentation of the stage due to the specific transition (i.e., transition frequency per unit time spent in the stage). Transition probability therefore could better reflect some element like a “pressure” to produce the transition than transition rate, and the “pressure” indicated by transition probability could reflect a relative difference among wake, REM, and non-REM (or N1, N2, and N3) sleep pressures. Therefore, overall, CFS patients could have less sleep pressure during REM sleep than controls, and this interpretation may relate to the common complaint of “unrefreshing sleep” in CFS; we have seen this reduction in REM sleep in the baseline condition (Kishi et al. 2008), and it remained reduced after exercise. While a literature exists showing evidence of the relationship between selective REM sleep disruption and daytime sleepiness in normal subjects (Glovinsky et al. 1990), such a relationship was not confirmed in patients with REM-predominant SDB (Chami et al. 2010), who have similar daytime manifestations to CFS patients such as fatigue, sleepiness, and cognitive impairment. This emphasizes the notion that disruption in REM may be a unique marker of the underlying pathophysiology of CFS. Our results further indicate there could be a spectrum in REM disruption even within patients with CFS – a result not seen in healthy controls. From a practical point of view, the results of these correlation analyses suggest transition probability may be a more useful metric than transition rate when predicting subjective feelings as a continuous variable.

One limitation of this study relates to the fixed sequence of events in the study design; this might lead to concerns that improved sleep on the later night in the sleep laboratory relative to the former night simply reflects habituation to being in the sleep laboratory. Based on the fact that exercise increased fatigue and decreased activity in CFS (Sisto et al. 1998; Ohashi et al. 2002; King and Jason 2005; Yoshiuchi et al. 2007), we did not employ a counterbalanced design to avoid the possible carry over effect of exercise. The fact that all subjects underwent an adaptation PSG prior to the baseline night, and our previous report of no difference in the traditional sleep variables, transition probabilities and ultradian REM sleep rhythm between the second and the third nights in the three nights spent in the laboratory (Kishi et al. 2011a) argues against any possible habituation effect. While the ideal way to deal with the habituation issue would have been to have adaptation studies before the baseline and exercise studies and to do a sham experiment with no exercise component, we simply could not follow this design due to problems with patients being unwilling to participate in multiple sleep studies.

Our use of 30 sec sleep stage scoring based on R&K (Rechtschaffen and Kales 1968) is another possible limitation. Although the advantage of doing this is that this scoring regimen is widely and easily applicable to existing data, the time resolution may not be good enough to capture fine microstructural changes of sleep such as arousals. Indeed, our continuity analysis noted two discrepant findings for CFS with respect to decreased nocturnal arousals after exercise: (1) less continuous N1 which is not consistent with decreased arousals and (2) more continuous REM which is consistent with decreased arousals. Incorporation of arousal information into the current transition analysis should be the next step to refine the analysis.

Another limitation has to do with the clinical population studied – one in which the diagnosis was made using clinical criteria, leading to a clinically heterogeneous population. We have tried to reduce heterogeneity by studying women only at a fixed time in their menstrual cycle and those who did not have evidence of sleep disorders or major depressive disorder. These choices obviously limit the generalizability of the data presented. The effects of exercise on sleep depend on various factors associated with the exercise task, including intensity, duration, and timing of the exercise. In this study, we used a standardized bout of maximal exercise, which was performed in the afternoon. Findings reported here may therefore be limited to these conditions, with further systematic research needed to thoroughly examine and establish the effects of exercise on sleep.

In conclusion, we have shown that exercise promotes transitions to deeper sleep stages and inhibits transitions to lighter sleep stages for both healthy controls and CFS patients. CFS patients however showed coexisting sleep disruption and more fatigue. While exercise had positive effects on dynamic sleep morphology in both healthy controls and CFS patients, CFS patients may not fully normalize their sleep with exercise alone. The observation of improved sleep despite worsening of symptoms in CFS after exercise needs to be further investigated. The specific sleep finding in CFS, increased transitions from REM to wake, could be a target of the treatment to improve the symptoms of fatigue, sleepiness, and pain in CFS.
